# Supplementary material for: Body Composition and “Catch-Up” Fat Growth in Healthy Small for Gestational Age Preterm Infants and Neurodevelopmental Outcomes
Source: Nutrients. 2022 Jul 25;14(15):3051. doi: 10.3390/nu14153051 (PMC9332383; doi:10.3390/nu14153051)
Supplement: Supplementary file 1 [file nutrients-14-03051-s001.zip › nutrients-1820455-supplementary.pdf]

Supplementary Materials

Table S1. Feeding Guidelines for Neonates 751-1000 gm Birth Weight.

Feeding Progression (16 Days)	Total Volume (mL/kg/day)	Feeding volume (mL) <u>every 3 hrs</u>	Caloric density (kcal/oz)	Additional Orders
1	**12**		20	
2	**12**		20	
3	**12**		20	
4	**12**		20	
5	**12**		20	
6	**21**<br>**29.5**		20	
7	**38.5**<br>**47**		20	
8	**56**<br>**64.5**		20	
9	**73.5**<br>**82**		20	
10	**90.5**<br>**100**		20	**D/C Lipids (p.m.)**
11	**100**		**22**<br>**24**	**Fortify* BM to 22 kcal/oz(1:50) (a.m.)**<br>**Fortify* BM to 24 kcal/oz(1:25) (p.m.)**
12	**108**<br>**117**		24	**Stop PN (p.m.)**
13	**125.5**<br>**134.5**		24	
14	**143**<br>**152**		24	
15	**160**		24	

*Fortification should be Similac Extensively Hydrolyzed Protein Liquid Fortifier.

1st Column: The 1st day of enteral feeds (BM or premature formula) that is given is "1/16" and so forth.

2nd Column: This column denotes the total volume of feedings per kg per day the pt will receive with eachadvance. Feeding advances will take place 12 hours apart for days with 2 advancing steps.

3rd Column: This column denotes the volume of feeding that is administered to the pt every 3 hours.

4th Column: This is the caloric density of the maternal or donor breast milk to be used that day.

5th Column: Note additional orders to be written for the patient on the appropriate day.

Notes: 1. The 1st day of feedings might not be the first day of life. Please beginfeedings when the medical team decides it is safe to do so.

2. Use birth weight until the patient has regained it; then use actual weight.

3. Administer the colostrum in the order it was pumped.

Table S2. Feeding Guidelines for Neonates ≤ 750 gm Birth Weight.

Feeding Progression (16 Days)	Total Volume (mL/kg/day)	Feeding volume (mL) every 3 hrs	Caloric density (kcal/oz)	Additional Orders
1	**12**		20	
2	**12**		20	
3	**12**		20	
4	**12**		20	
5	**12**		20	
6	**20**<br>**27**		20	
7	**34.5**<br>**42**		20	
8	**49.5**<br>**57**		20	
9	**64.5**<br>**72**		20	
10	**80**<br>**87**		20	
11	**94.5**<br>**102**		20	**D/C Lipids (p.m.)**
12	**102**		**22**<br>**24**	**Fortify* BM to 22 kcal/oz(1:50) (a.m.)**<br>**Fortify* BM to 24 kcal/oz(1:25) (p.m.)**
13	**109.5**<br>**117**		24	**Stop PN (p.m.)**
14	**124.5**<br>**132**		24	
15	**139.5**<br>**147**		24	
16	**154.5**<br>**160**		24	

*Fortification should be Similac Extensively Hydrolyzed Protein Liquid Fortifier.

1st Column: The 1st day of enteral feeds (BM or premature formula) that is given is "1/17" and so forth.

2nd Column: This column denotes the total volume of feedings per kg per day the pt will receive with eachadvance. Feeding advances will take place 12 hours apart for days with 2 advancing steps.

3rd Column: This column denotes the volume of feeding that is administered to the pt every 3 hours.

4th Column: This is the caloric density of the maternal or donor breast milk to be used that day.

5th Column: Note additional orders to be written for the patient on the appropriate day.

Notes: 1. The 1st day of feedings might not be the first day of life. Please beginfeedings when the medical team decides it is safe to do so.

2. Use birth weight until the patient has regained it; then use actual weight.

3. Administer the colostrum in the order it was pumped.

Table S3. Feeding Guidelines for Neonates 1001-1250 gm Birth Weight.

Feeding Progression (11 days)	Total Volume (mL/kg/day)	Feeding Volume (mL) every 3 hrs	Caloric density (kcal/oz)	Additional Orders
1	**12**		20	
2	**12**		20	
3	**12**		20	
4	**24.5**<br>**37**		20	
5	**49.5**<br>**62**		20	
6	**74.5**<br>**87**		20	
7	**99.5**<br>**112**		20	**D/C Lipids (p.m.)**
8	**112**		**22**<br>**24**	**Fortify* BM to 22 kcal/oz (1:50)**<br>**Fortify* BM to 24 kcal/oz (1:25)**
9	**124.5**<br>**137**		24	**Stop PN (p.m.)**
10	**149.5**<br>**160**		24	

*Fortification should be Similac Extensively Hydrolyzed Protein Liquid Fortifier.

1st Column: The 1st day of enteral feeds (BM or premature formula) that is given is "1/11" and so forth.

2nd Column: This column denotes the total volume of feedings per kg per day the pt will receive with eachadvance. Feeding advances will take place 12 hours apart for days with 2 advancing steps.

3rd Column: This column denotes the volume of feeding that is administered to the pt every 3 hours.

4th Column: This is the caloric density of the maternal or donor breast milk to be used that day.

5th Column: Note additional orders to be written for the patient on the appropriate day.

Notes: 1. The 1st day of feedings might not be the first day of life. Please beginfeedings when the medical team decides it is safe to do so.

2. Use birth weight until the patient has regained it; then use actual weight.

3. Administer the colostrum in the order it was pumped.

Table S4. Feeding Guidelines for Neonates 1251-1500 gm Birth Weight.

Feeding Progression (8 days)	Total Volume (mL/kg/day)	FeedingVolume (mL) every 3 hrs	Caloric density (kcal/oz)	Additional Orders
1	**12**		20	
2	**27**<br>**42**		20	
3	**57**<br>**72**		20	
4	**87**<br>**102**		20	**D/C lipids (p.m.)**
5	**102**		**22**<br>**24**	**Fortify* BM to 22 kcal/oz (1:50)**<br>**Fortify* BM to 24 kcal/oz (1:25)**
6	**117**<br>**132**		24	**Stop PN**
7	**147**<br>**160**		24	

*Fortification should be Similac Extensively Hydrolyzed Protein Liquid Fortifier.

1st Column: The 1st day of enteral feeds (BM or premature formula) that is given is "1/8" and so forth.

2nd Column: This column denotes the total volume of feedings per kg per day the pt will receive with eachadvance. Feeding advances will take place 12 hours apart for days with 2 advancing steps.

3rd Column: This column denotes the volume of feeding that is administered to the pt every 3 hours.

4th Column: This is the caloric density of the maternal or donor breast milk to be used that day.

5th Column: Note additional orders to be written for the patient on the appropriate day.

Notes: 1. The 1st day of feedings might not be the first day of life. Please beginfeedings when the medical team decides it is safe to do so.

2. Use birth weight until the patient has regained it; then use actual weight.

3. Administer the colostrum in the order it was pumped.

Table S5. Feeding Guidelines for Neonates 1501-2000 gm Birth Weight.

Feeding Progression (8 days)	Total Volume (mL/kg/day)	FeedingVolume (mL) every 3 hrs	Caloric density (kcal/oz)	Additional Orders
1	**30**		20	
2	**47.5**<br>**65**		20	
3	**82.5**<br>**100**		20	**D/C lipids (p.m.)**
4	**100**		**22**<br>**24**	**Stop PN (p.m.)**<br>**Fortify* BM to 22 kcal/oz (1:50)**<br>**Fortify* BM to 24 kcal/oz (1:25)**
5	**117.5**<br>**135**		24	
6	**152.5**<br>**160**		24	

*Fortification should be Similac Concentrated Liquid Fortifier.

1st Column: The 1st day of enteral feeds (BM or premature formula) that is given is "1/6" and so forth.

2nd Column: This column denotes the total volume of feedings per kg per day the pt will receive with eachadvance. Feeding advances will take place 12 hours apart for days with 2 advancing steps.

3rd Column: This column denotes the volume of feeding that is administered to the pt every 3 hours.

4th Column: This is the caloric density of the maternal or donor breast milk to be used that day.

5th Column: Note additional orders to be written for the patient on the appropriate day.

Notes: 1. The 1st day of feedings might not be the first day of life. Please beginfeedings when the medical team decides it is safe to do so.

2. Use birth weight until the patient has regained it; then use actual weight.

3. Administer the colostrum in the order it was pumped.

The NICU Enteral Feeding Guidelines

Carrie Finch, MS, RD, LD and CNSC Medical University of South Carolina

There are five feeding guidelines that were developed to achieve a more consistent approach to the feeding of our preterm neonates. These guidelines offer recommendations regarding the volume of each feeding, when to increase feeding volumes, and when to fortify the milk to 24 kcals/ounce[6,9]. The 1st day of feedings might not be the first day of life. Please begin feedings when the medical teamdecides it is safe to do so and places an order on the chart. When feedings begin to advance in volume, volume increases should be made 12 hours apart. Colostrum should be administered in the order it was pumped. **These guidelines were not developed to replace clinical judgement or to eliminate individualizing the feeding plan to meet unique patient needs.**

Minimal Enteral Nutrition (MEN) or Trophic Feedings:

Minimal Enteral Nutrition (MEN), also known as trophic feedings, are small volume enteral feedings (< 15-20 mL/kg/d) given within 6-24 hours of life for several days to the previously unfed premature infant.MEN enhances mucosal maturity, gut motility, and stimulates secretion of intestinal hormones. For these reasons, MEN may shorten the time to achieve full EN and possibly decrease the risk of necrotizing enterocolitis[4,5,10-12].

Contraindications to Minimal Enteral Nutrition (MEN)[8]:

1. Requirement for cardiovascular pharmacological support (i.e., pressors or PGEs)
2. Significant PPHNS
3. Surgical abdomen
4. Inability to maintain minimally acceptable oxygenation

Signs of Feeding Intolerance[8,12]:

1. Grossly bloody stools
2. Emesis (includes bilious and non-bilious)
3. Abnormal physical exam (including umbilical or abdominal discoloration, tight distention, glisteningabdomen, tenderness)

-If abdominal exam is abnormal (prior to each feeding), notify MD or NNP.

Management of Residuals[3,6]:

Trophic Feedings:

Volume of Residuals (with normal physical exam):

< 2 mL → Refeed residual and give this feeding

> 2 mL → Discard residual and continue with feedingsGreen Residuals:

Confirm placement of the NG/OG tube (to ensure it is not transpyloric)

During trophic feedings, green residuals are generally related to immature or paradoxical gut motility and **by themselves** are not indicative of feeding intolerance.If accompanied by other signs of feeding intolerance (see above) → **notify MD/NNP.**

Bloody Residuals:

require notification of MD/NNP

Feeding Advancement:

Volume of Residuals:

Residuals with an abnormal physical exam or clinical signs of feeding intolerance→**Notify MD/NNP**

≤ 50% of the feeding volume (normal physical exam)→proceed with feedings

> 50% of the feeding volume→evaluate for signs of feeding intolerance **and** notify MD/NNP

Green or Bloody Residuals:

require notification of MD/NNP

Parenteral Nutrition (PN) Criteria[8,10]:

Neonates with birth weights ≤ 1800 gm would automatically be candidates for PN and qualify for the "After Hours" (aka "DOL 1") bag. These patients should be placed on this until PN can be ordered forthe next day.

Patients should receive PN if:

1. Anticipated NPO > 48 hours (as in congenital malformations, GI malformations, PDA withIndocin therapy or awaiting ligation, PPHN, pressor requirement for cardiovascular instability). Consider initiate an "After Hours" PN for these infants.
2. Patients with continued NPO status on the 3rd day of life
3. Failed enteral feeding advancement plan

Birth weight ≤ 1800 grams

Beneprotein Supplement:

In order to achieve at least 3.5 grams/kg/day of protein in our VLBW infants receiving human milk, we piloted 2 feeding algorithms. The first algorithm increased enteral feeding volumes to 180 cc/kg/day of fortified human milk (24 kcal/oz) using human milk fortifier (1packet : 25 cc's HM) which provided 144 kcal/kg and 3.6 g pro/kg (protein/energy ratio of

2.5 g per 100 kcal). After piloting 10 patients, this trial was considered a failure as all but 2patients were not able to tolerate the higher volume from a respiratory standpoint and weredecreased back to 160 cc/kg/day.

The second algorithm used a goal enteral volume of 160 cc/kg/day (our nursery standard). Beneprotein® was added when goal volume was achieved. The amount of Beneprotein® added was ¼ teaspoon to 50 cc's FHM for an additional 0.65 g pro/dL (protein/energy ratio of 3.2 g per 100 kcal). If growth velocity remained < 18 g/kg/day after 48-72 hours, the Beneprotein® supplementation was increased to give an additional 1.3 g pro/dL (¼ teaspoonto 25 cc's FHM). At 160 cc/kg/day, this provided 139 kcal/kg and ~5.3 g pro/kg (protein/energy ratio of 3.8 g per 100 kcal). After piloting 10 patients with this algorithm, it was found that 100% of the patients had Beneprotein® increased to ¼ teaspoon to 25 cc FHM. The pilot data was presented to the Neonatology division where it was decided to standardize protein supplementation as ¼ teaspoon to 25 cc FHM to be added when VLBW infants achieve 160 cc/kg/day of FHM feedings. The pilot also found there to be no feeding intolerance associated with the addition of Beneprotein®, nor any lab abnormalities (ie., BUN, creatinine). This change was implemented into our automated enteral feeding advancement orders and began on 7/14/10.

References

1. Caple J, Armentrout D, Huseby V, Halbardier B, Garcia J, Sparks JW, Moya FR. Randomized, controlled trial of slow versus rapid feeding volume advancement in preterm infants. *Pediatrics*. 2004; 114:1597-1600.
2. Cobb BA, Waldemar CA, Ambalavanan N. Gastric residuals and their relationship to necrotizingenterocolitis in very low birth weight infants. *Pediatrics*. 2004; 113(1): 50-3
3. Kennedy KA, Tyson JE. Rapid versus slow rate of advancement of feedings for promoting growth and
4. preventing necrotizing enterocolitis in parenterally fed low birth weight infants (Review). *Cochrane Database ofSystematic Reviews*. 2005;4:1-11.
5. Mishra S, Agarwal R, Jeevasankar M, Deorari AK, Paul VK. Minimal enteral nutrition. *Indian J Pediatrics*. 2008;75: 267-9.
6. Mosqueda E, Sapiegiene L, Glynn L, Wilson-Costello D, Weiss M. The use of minimal enteral nutrition in extremely lowbirth-weight newborns. *J Perinatol*. 2008;28: 264-9.
7. Premji SS, Paes B, Jacobson K, Chessell L. Evidence-based feeding guidelines for very low-birthweight infants. *Advancesin Neonatal Care*. 2002;2(1): 5-18.
8. Rayyis SF, Ambalavanan N, Wright L, Carlo WA. Randomized trial of "slow" versus "fast" feed
9. advancements on the incidence of necrotizing enterocolitis in very low birth weight infants. *J Pediatrics*. 1999;134(3).

10. Reiter PD, Thureen PJ. Nutrition support in neonatology. In: Gottschlich, editor. The science andpractice of nutrition support. Dubuque: Kendall/Hunt Publishing Company, 2001:325-33.
11. Street JL, Montgomery D, Alder SC, Lambert DK, Gerstmann DR, Christensen RD. Implementing feeding guidelines forNICU patients < 2000 g results in less variability in nutrition outcomes. *JPEN*. 2006;30(6): 515-18.
12. Simmer K. Aggressive nutrition for preterm infants—benefits and risks. *Early Human Development*. 2007;83: 631-4.
13. Tyson JE, Kennedy KA. Trophic feedings for parenterally fed infants (Review). *Cochrane Database of SystematicReviews*. 2005;3: 1-21.
14. Yu V, Simmer K. Enteral nutrition: practical aspects, strategy and management. In: Tsang RC, Uauy
15. R, Koletzko B, Zlotkin SH, eds. Nutrition of the preterm infant: scientific basis and practical guidelines. 2nd edition.Cincinnati: Digital Educational Publishing, Inc., 2005: 311-32.
